# Machine learning nominates the inositol pathway and novel genes in Parkinson’s disease

**DOI:** 10.1093/brain/awad345

**Published:** 2023-10-06

**Authors:** Eric Yu, Roxanne Larivière, Rhalena A Thomas, Lang Liu, Konstantin Senkevich, Shady Rahayel, Jean-François Trempe, Edward A Fon, Ziv Gan-Or

**Affiliations:** Department of Human Genetics, McGill University, Montreal, Quebec H3A 0G4, Canada; The Neuro (Montreal Neurological Institute-Hospital), Montreal, Quebec H3A 2B4, Canada; Department of Neurology and Neurosurgery, McGill University, Montreal, Quebec H3A 0G4, Canada; Department of Neurology and Neurosurgery, McGill University, Montreal, Quebec H3A 0G4, Canada; Early Drug Discovery Unit (EDDU), Montreal Neurological Institute-Hospital (The Neuro), Montreal, Quebec H3A 2B4, Canada; Department of Human Genetics, McGill University, Montreal, Quebec H3A 0G4, Canada; The Neuro (Montreal Neurological Institute-Hospital), Montreal, Quebec H3A 2B4, Canada; The Neuro (Montreal Neurological Institute-Hospital), Montreal, Quebec H3A 2B4, Canada; Department of Neurology and Neurosurgery, McGill University, Montreal, Quebec H3A 0G4, Canada; Centre for Advanced Research in Sleep Medicine, Hôpital du Sacré-Cœur de Montréal, Montreal, Quebec H4J 1C5, Canada; Department of Medicine, University of Montreal, Montreal, Quebec H3C 3J7, Canada; Department of Pharmacology and Therapeutics and Centre de Recherche en Biologie Structurale, McGill University, Montreal, Quebec H3A 0G4, Canada; Department of Neurology and Neurosurgery, McGill University, Montreal, Quebec H3A 0G4, Canada; Early Drug Discovery Unit (EDDU), Montreal Neurological Institute-Hospital (The Neuro), Montreal, Quebec H3A 2B4, Canada; Department of Human Genetics, McGill University, Montreal, Quebec H3A 0G4, Canada; The Neuro (Montreal Neurological Institute-Hospital), Montreal, Quebec H3A 2B4, Canada; Department of Neurology and Neurosurgery, McGill University, Montreal, Quebec H3A 0G4, Canada

**Keywords:** Parkinson’s disease, GWAS, machine learning, gene prioritization

## Abstract

There are 78 loci associated with Parkinson’s disease in the most recent genome-wide association study (GWAS), yet the specific genes driving these associations are mostly unknown. Herein, we aimed to nominate the top candidate gene from each Parkinson’s disease locus and identify variants and pathways potentially involved in Parkinson’s disease. We trained a machine learning model to predict Parkinson’s disease-associated genes from GWAS loci using genomic, transcriptomic and epigenomic data from brain tissues and dopaminergic neurons. We nominated candidate genes in each locus and identified novel pathways potentially involved in Parkinson’s disease, such as the inositol phosphate biosynthetic pathway (*INPP5F*, *IP6K2*, *ITPKB* and *PPIP5K2*). Specific common coding variants in *SPNS1* and *MLX* may be involved in Parkinson’s disease, and burden tests of rare variants further support that *CNIP3*, *LSM7*, *NUCKS1* and the polyol/inositol phosphate biosynthetic pathway are associated with the disease. Functional studies are needed to further analyse the involvements of these genes and pathways in Parkinson’s disease.

## Teaser

Yu *et al*. train a machine learning model to predict Parkinson’s disease-associated genes from GWAS loci using genomic, transcriptomic and epigenomic data from brain tissues and dopaminergic neurons. They propose candidate genes for each locus, and identify novel pathways that may be involved in Parkinson’s disease.

**See Lanore *et al.* (https://doi.org/10.1093/brain/awae043) for a scientific commentary on this article**.

## Introduction

Genome-wide association studies (GWAS) have nominated many variants associated with complex traits. In Parkinson’s disease (PD), the most recent GWAS revealed 90 independent risk variants across 78 genomic loci.^1^ Although many single-nucleotide polymorphisms (SNPs) are in novel genomic loci, well-established PD genes discovered many years ago, such as *LRRK2*, *PINK1*, *DJ-1*, *SNCA*, *GBA1*, *PRKN* and *MAPT* still account for the vast majority of research on this disease.

Several disadvantages of GWAS limit additional functional analyses. First, over 90% of all GWAS significant SNPs are in non-coding regions.^2^ These SNPs are often passenger variants due to complex linkage disequilibrium (LD). Second, the causal gene associated with the causal SNPs remains unclear in most GWAS loci.^3^ To overcome these challenges, downstream GWAS analyses were established with the aim of identifying causal genes within GWAS loci. This involves techniques such as fine-mapping and co-localization methods to nominate causal SNPs, as well as transcriptome-wide association studies to nominate gene-trait associations.^4-6^ These models use LD structure, and gene expression panels to discover causal SNPs/genes. While these methods may propose causal variants and genes, additional biological evidence is generally required to pair causal variants with causal genes. Using multi-omic analyses, one can integrate a diverse range of comprehensive cellular and biological datasets such as genomic, transcriptomic and epigenetic datasets and use platforms such as Open Targets Genetics (https://genetics.opentargets.org/) to perform systematic analyses of gene prioritization across all publicly available GWASs.^7^ Although powerful, Open Targets Genetics lacks disease-specific tissues relevant to PD such as dopaminergic neurons and microglia. Using a similar approach, we may discover additional pathways and genetic targets involved in PD.

**Figure 1:**
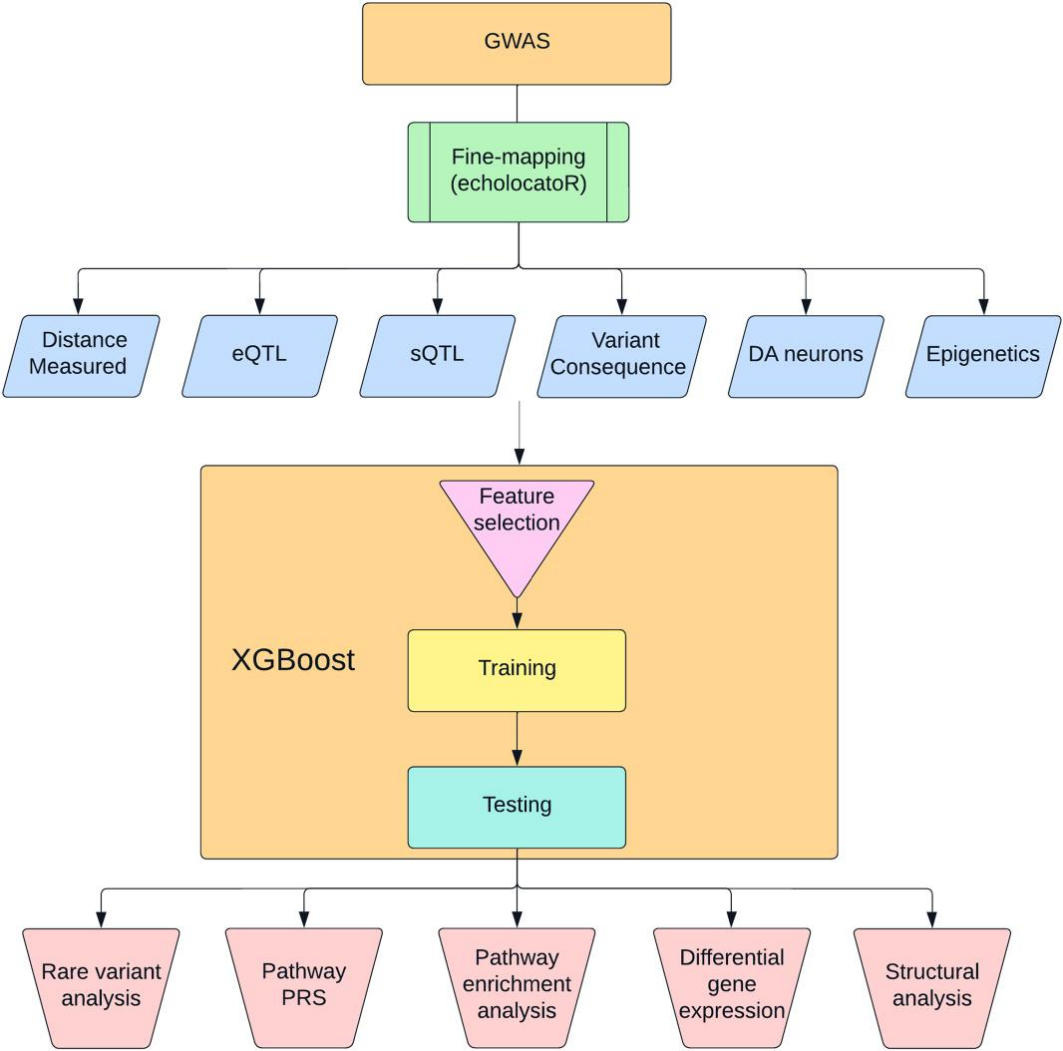
**Workflow summary.** This figure describes the analyses performed in this study. DA = dopaminergic; eQTL/sQTL = expression/splicing quantitative trait loci; GWAS = genome-wide association study; PRS = polygenic risk score.

In this study, we leveraged PD-relevant transcriptomic, epigenomic and other datasets in our gradient boosting model (Fig. 1). We trained this model on well-established PD genes to nominate causal genes from PD GWAS loci.

## Materials and methods

### General design of the study

Our objective was to nominate the most probable genes to be involved in PD from each GWAS locus based on the most recent PD GWAS (see Fig. 1 for the study protocol).^1^ To do so, we first defined all the genes and SNPs that are within these loci (see later) and used to a machine learning approach to nominate the top genes in each locus. Based on the previous literature and consensus between authors, we identified seven genes from well-established loci associated with PD that can be considered the likeliest driving genes of their respective loci (*GBA1*, *LRRK2*, *SNCA*, *GCH1*, *MAPT*, *TMEM175* and *VPS13C*). We then acquired data for multiple features, including different distance measures from top SNPs, different QTLs, expression in relevant tissues and cell types and predictions of variant consequences (78 features out of 284 were used after removal of redundant features; Supplementary Table 1). Using the seven well-established PD genes, which were labelled as positive, and 212 genes in the same loci that received negative labels (i.e. not likely to drive the association with PD, since the PD-driving gene is already well-established), we trained a machine learning model. This model enabled us to generate a prediction score for each gene within each locus, assessing their potential involvement in PD. The gene with the highest score in each locus is the nominated gene to be associated with PD. We then performed multiple *post hoc* analyses to further validate and explore our results: burden tests for rare variants in the top-scoring genes, pathway enrichment and pathway PRS analyses, differential expression analyses and structural analyses for candidate coding variants.

### Definition of loci and genes within each locus

Following the definition by Nalls *et al*.,^1^ all loci were defined based on the 90 independent risk variants (Supplementary Table 2). Variants within 250 kb were merged into a single locus, which led to 78 loci. All protein coding genes within 1 Mb of the risk variants were included in the model. To exclude non-causal variants, echolocatoR was used as a comprehensive fine-mapping model.^5^ This method leverages Bayesian statistical and functional fine-mapping tools as well as epigenomic data to calculate the causal probability of SNPs in a locus.^5^ In our downstream analysis, we incorporated the SNPs nominated by echolocatoR into the credible gene sets generated by the same tool. Furthermore, we included the 90 independent SNPs obtained from the PD GWAS in our analysis.

### Feature preprocessing

To leverage multi-omic data for the machine learning algorithm, we integrated a comprehensive list of datasets (Supplementary Table 1), which included SNP functional annotations, expression and splicing quantitative trait loci (eQTL/sQTL), single nuclear RNA sequencing (scRNA) and chromatin interaction. Since distance was previously shown to be the most predictive feature in about 60–70% of GWAS loci, the distances from each SNP to each gene in the locus and the distance to the transcription start site were included in the model.^8^ To predict the severity of variant consequences, we used VEP^9^ and PolyPhen-2.^10^ The SNP2GENE function on the FUMA platform was used to perform functional mapping of SNPs to eQTLs.^11^ In the FUMA settings, we chose the UK Biobank release2b 10k European reference panel, a maximum distance of 1000 kb from SNPs to gene, and included the major histocompatibility complex (MHC) region. All other FUMA settings were kept as default. Expressions QTL and 3D chromatin interaction mapping were performed using brain tissues, whole blood, Functional Annotation of the Mammalian genome (FANTOM) and Genotype-Tissue Expression (GTEx) datasets. Using scRNA datasets from Kamath *et al*.,^12^ we included gene expression from all ten subpopulations of dopaminergic neurons from post-mortem brains of seven PD and eight control donors. A complete list of all datasets can be found in Supplementary Table 3.

### Neighbourhood scores

To integrate the concept of locus and LD in the model, we calculated the neighbourhood scores for each feature by transforming the data relative to the best-scoring gene within each locus,^7^ allowing the model to find the highest expressed genes across each locus. For example, if the feature is ‘maximum gene expression in blood’, the gene with the highest expression in each locus would have a score of 1 while the score of the remaining genes in the locus would be calculated following the expression of gene divided by the expression of highest expressed gene in the locus. Negative log transformation was applied so that the closest gene had the highest score.

### Machine learning model to prioritize genes

We used XGBoost^13^ to train the machine learning model. We selected well-established genes from PD loci for the training dataset (*GBA1*, *GCH1*, *LRRK2*, *MAPT*, *SNCA*, *TMEM175*, *VPS13C*). These genes were labelled as positive labels, and the remaining genes from these same loci were labelled as negative labels. In total, the training set was composed of 212 genes (seven positive labelled and 205 negative labelled). The scale_pos_weight parameter in XGBoost was set to the ratio of negative to positive labels to control for the imbalance. The training process involved two steps. First, we performed feature selection to detect redundant features. This involved removing any variables from the dataset that were either redundant or uninformative. XGBoost was employed to transform the dataset into a subset containing the chosen features. To achieve this, we trained a model using the complete dataset and then retained the features present in the subset produced by XGBoost. In the second step, the final training model was created using the selected features. This two-step approach helps optimize the training process and ensures that the model focuses on relevant and informative features to make accurate predictions. We performed hyperparameter tuning and 5-fold cross-validation on both models. Mean average precision was used as an evaluation function to maximize the score of correct positive predictions made. Of the 284 features, 78 features passed feature selection for the final training model.

### Functional enrichment analysis

To examine whether specific pathways may be involved in PD, based on the genes nominated in each locus, we performed an over-representation analysis using WebGestalt (Web-based Gene Set Analysis Toolkit) on 25 January 2023.^14^ We included the top candidate gene from each locus, and examined enrichment in terms of biological processes and cellular components from the Gene Ontology (GO) data. The genome protein-coding list was used as the reference list and pathways were considered to be associated with PD if significant after false discovery rate (FDR) correction.

### Single-cell and bulk RNA sequencing analyses

To examine whether genes nominated by the machine learning model may be differentially expressed in PD relevant models, we used publicly available single-cell and bulk RNA sequencing (RNAseq) data from The Foundational Data Initiative for Parkinson’s disease (FOUNDIN-PD)^15^ and Kamath *et al*.^12^ FOUNDIN-PD scRNA data include 80 induced pluripotent stem cell (iPSC) lines collected after 65 days.^15^ We then performed differential gene expression analyses between PD cases and controls. For scRNA, we used the MAST^16^ package after adjusting for covariates, such as age, sex and batch. For bulk RNAseq, we used DESeq2,^17^ while adjusting for the same covariates.

### Pathway polygenic risk score analyses

Pathway-specific polygenic risk score (PRS) analysis can further support a role for specific pathways in PD.^18^ Using PRSet,^19^ pathway-specific PRSs were calculated for pathways nominated by gene set analysis on 14 828 PD cases and 13 283 controls from seven cohorts [McGill, Parkinson’s Progression Markers Initiative (PPMI), Vance (dbGap phs000394), International Parkinson’s Disease Genomics Consortium (IPDGC) NeuroX dataset (dbGap phs000918.v1.p1), National Institute of Neurological Disorders and Stroke (NINDS) Genome-wide genotyping in Parkinson’s disease (dbGap phs000089.v4.p2), NeuroGenetics Research Consortium (NGRC) (dbGap phs000196.v3.p1) and UK Biobank]. The number of cases and controls for each cohort is described in Supplementary Table 4. Participants were unrelated individuals of European ancestry and were not gender matched. Rare SNPs (minor allele frequency < 0.01) with a *P*-value < 0.05 were excluded from the analysis. LD clumping was performed using *r*^2^ = 0.1 and 250 kb distance. Permutation testing was performed with 10 000 label permutations to generate an empirical *P*-value for each gene set after adjusting for a prevalence of 0.005, age at onset for cases, age at enrollment for control, sex and the top 10 principal components. The Vance cohort was excluded from the meta-analysis due to significant heterogeneity.

### Rare variant burden analyses

To examine whether there is an association between rare variants in the genes nominated by the machine learning model and PD, we used MetaSKAT^20^ to perform a meta-analyses of rare variants. We used whole exome sequencing (WES) available for 602 PD patients, 6284 proxy patients and 140 207 controls from UK Biobank (*n* = 147 093) and 2600 PD patients, 3677 controls from Accelerating Medicines Partnership Parkinson’s Disease (AMP-PD)^21^ datasets (*n* = 6277). Additional selection criteria for UK Biobank and AMP PD were reported previously.^22,23^ We performed the analysis on several groups of rare variants (allele frequency < 0.01): loss of function variants; non-synonymous variants; potentially deleterious (CADD > 20) variants; and functional (including non-synonymous, frame-shift, stop-gain and splicing) variants. Pathway-specific rare variant analysis was performed by combining PD genes from the pathways nominated previously. All analyses were adjusted for age at onset for cases, age at sample for controls and sex.

### Structural analysis

The atomic coordinates of SPNS1 (UniProt #H3BR82) were retrieved from the AlphaFold server (https://alphafold.ebi.ac.uk/). The structures of MLX-MAD1 and MLX-MLXIP were generated using AlphaFold-Multimer version 3, as implemented in ColabFold.^24,25^ The ternary complex with a DNA duplex was generated by superposing the heterodimers on the crystal structure of the MAD1-MAX-DNA complex (PDB 1NLW). The figures were generated using PyMol v.2.4.0.

## Results

### Machine learning model nominates PD-associated genes in each PD locus

**Figure 2:**
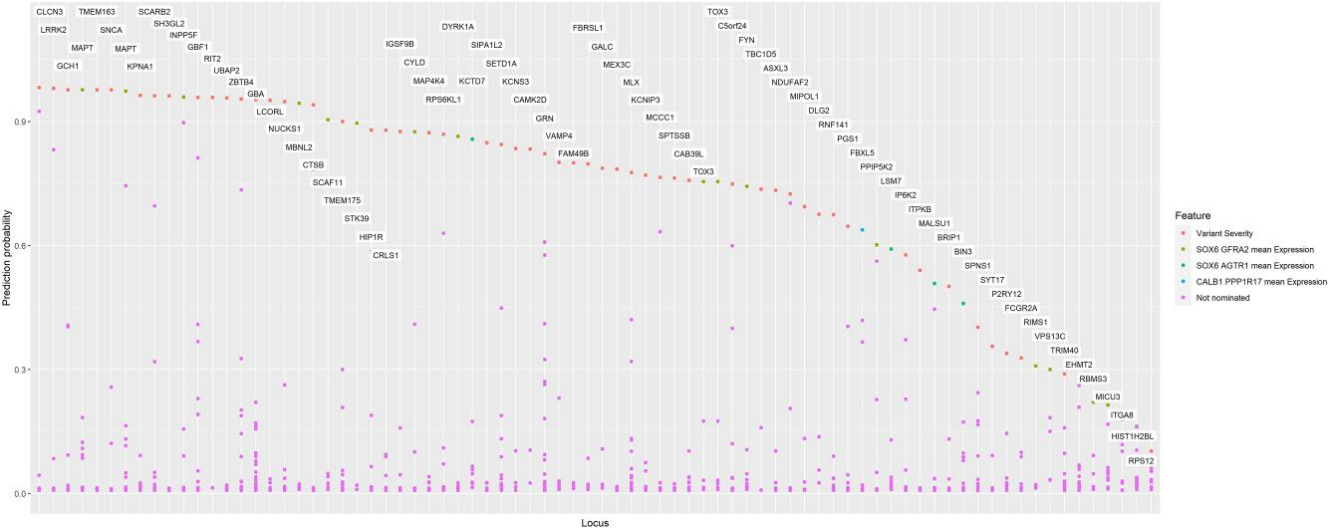
**Probability score of the Parkinson’s disease genome-wide association study candidate genes.** This figure shows the probability scores from the machine learning model for each locus in the Parkinson’s disease genome-wide association study loci sorted in descending order. For each gene, the top non-distance feature was used to colour the data.

To train our machine learning model, we used seven well-established PD-associated genes from the PD GWAS (*GBA1*, *LRRK2, SNCA, GCH1*, *MAPT*, *TMEM175* and *VPS13C*) as positive labels, and the remaining genes from the same loci (*n* = 205) were used as negative labels (i.e. genes that are unlikely to be involved in PD). We trained an XGBoost regression model to identify the best predictive features. Then, based the best predictive features, we assigned a probability score that indicated the likelihood that the gene was driving the association at each locus (Supplementary Table 2). We then nominated the top-scoring genes in each locus (Fig. 2 and Supplementary Table 2). Two genes, *MAPT* and *TOX3*, were nominated twice in neighbouring loci that harbour them, taking the total number of genes nominated in this model to 76 genes in 78 loci. A probability score higher than 0.75 was assigned to 48 of the 76 genes (63%). Of note, five genes (*NEK1*, *FDFT1*, *PSD*, *BAG3* and *SLC2A13*) that were ranked second in their respective loci also had a probability score >0.75. However, the nominated genes in their loci (*CLCN3*, *CTSB*, *GBF1*, *INPP5F* and *LRRK2*, respectively) all had probability scores >0.94. In seven other loci, the top nominated genes had an especially low probability score (<0.3), including *RBMS3*, *HIST1H2BL*, *TRIM40*, *EHMT2*, *RPS12*, *MICU3* and *ITGA8*.

### Gene expression in subtypes of PD-associated dopaminergic neurons predicts PD-relevant genes

**Figure 3:**
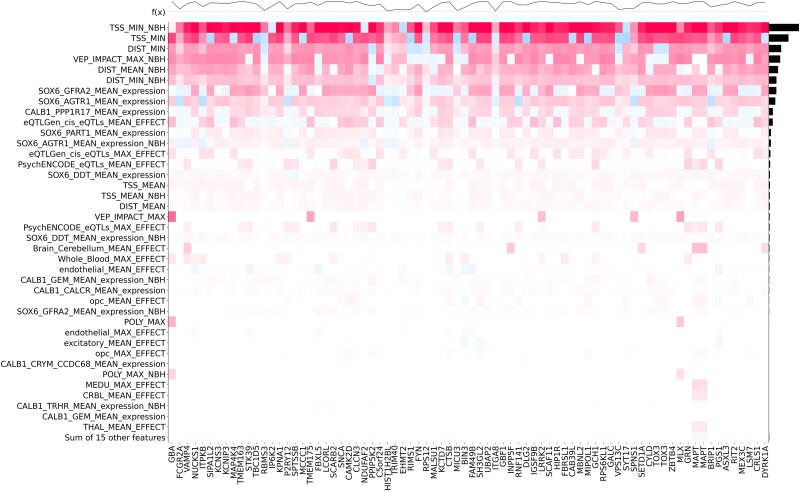
**Feature importance for the Parkinson’s disease genome-wide association study gene prioritization model.** Bee-swarm plot of feature importance using Shapley Additive exPlanations values along with the distribution of genes based on feature value.

**Figure 4:**
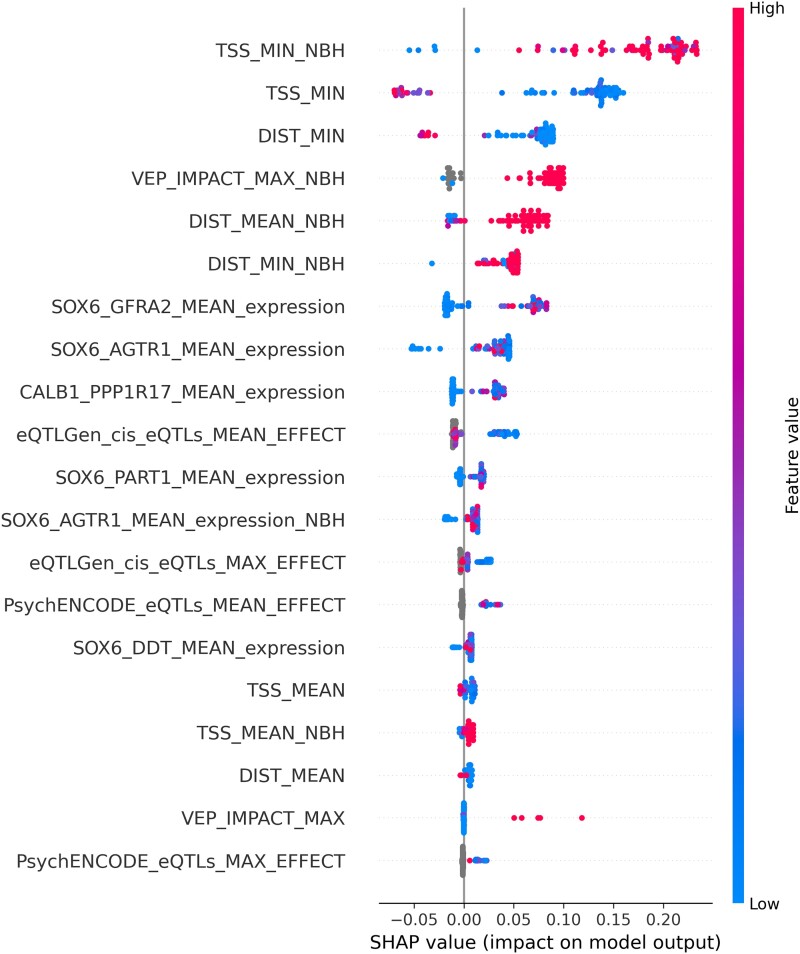
**Heat map of feature importance.** The heat map is generated using Shapley Additive exPlanations (SHAP) value for the top candidate gene in each locus. The plot at the top represents the probability score of each gene.

**Figure 5:**
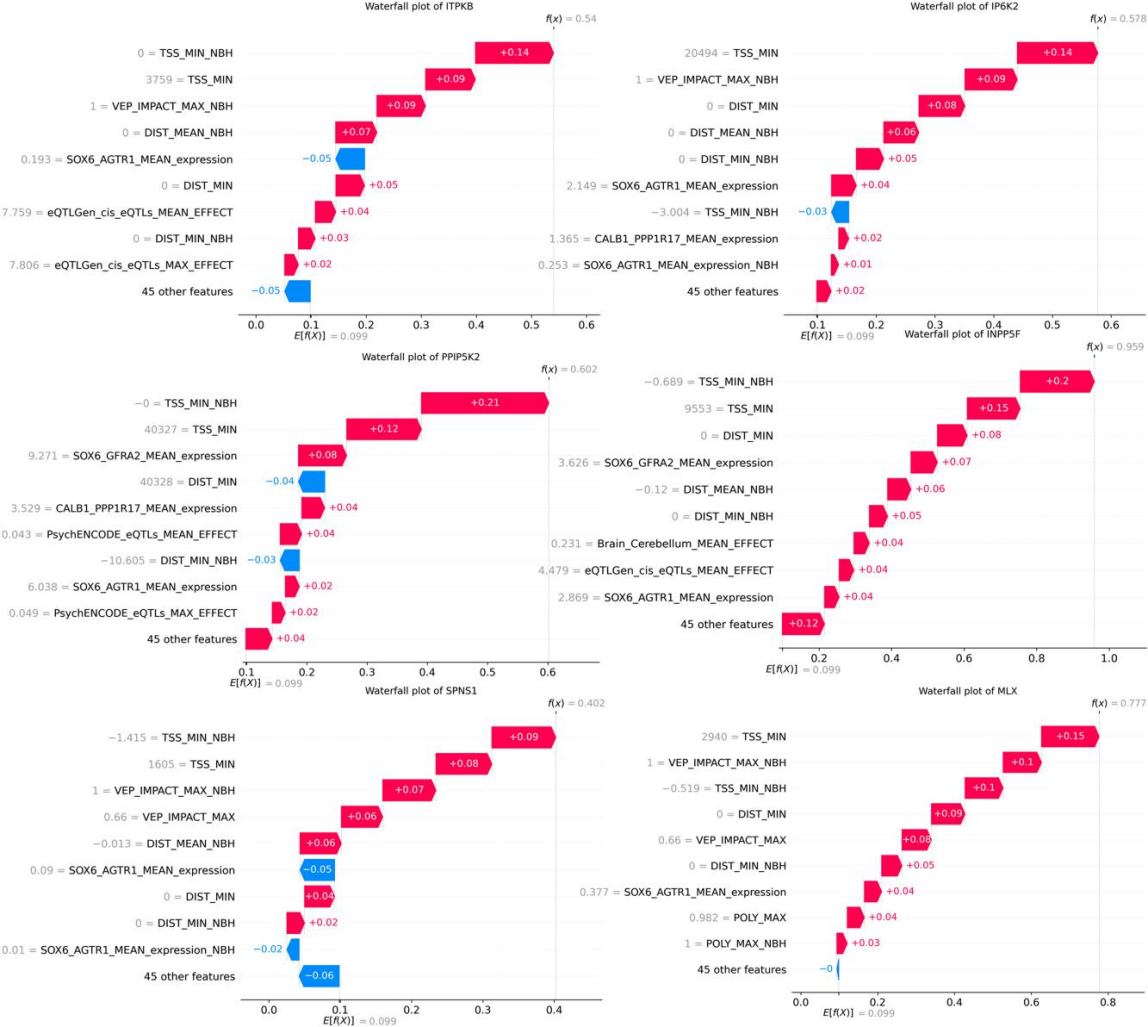
**Waterfall plots for Parkinson’s disease genome-wide association study candidate genes.** Importance of the top 10 features using Shapley Additive exPlanations values for different selected candidate genes. E[f(x)] is the base score for each gene, which is calculated based on the average value of each features. f(x) is the final score after accounting for all features.

Next, we used Shapley Additive exPlanations (SHAP) values to determine which features of the model contributed most to the prediction.^26,27^ SHAP values provide, for each gene, the relative contribution of each feature to the selection of that gene. The most important features for the scoring of each gene are shown in Fig. 3. As expected, distance-related features, such as distance from the top-associated SNP in the locus to the transcription start site or distance to the beginning of the gene, were the most important features in our model.^7^ The next most important feature was the Variant Effect Predictor (VEP) value, followed by additional distance measures.^9^ Interestingly, the next most important features were mRNA expression values within specific dopaminergic neuron subtypes. These different dopaminergic neuron subtypes are defined by the expression of the genes *GFRA2* and *AGTR1* from single nuclear sequencing of post-mortem tissue. The latter is a specific subtype of dopaminergic neurons shown by Kamath *et al*.^12^ to be selectively degenerated in brains of PD patients.^12^ The remaining features include expression in other dopaminergic neuron subpopulations, eQTLs and other expression features. Epigenetic features were not predictive in our model. As shown in Fig. 4, all nominated genes had at least one of the distance features contributing to their selection. On top of the known contribution of missense variants in *GBA1*, *LRRK2* and *GCH1*, we nominated missense SNPs that contributed to the score of two candidate genes: *SPNS1* (p.L512M, rs7140) and *MLX* (p.Q139R, rs665268). In Europeans, both SNPs are in high LD with the candidate GWAS SNPs of their respective locus (*SPNS1* D': 0. 88 *r*^2^: 0.74; *MLX* D': 1 *r*^2^: 1). In GTEx, rs7140 and rs665268 are also eQTLs/sQTLs for *SPNS1* and *MLX* across several PD related tissues such as whole blood and anterior cingulate cortex. The eQTL and sQTL results from GTEx v8 are shown in Supplementary Table 5. *SPNS1* and *MLX* have not previously been implicated in PD, and the important features identifying these genes as the top candidate for their respective GWAS loci are shown in Fig. 5.

### Differential expression of genes from the inositol phosphate biosynthetic pathway and *MLX* in PD

To further establish the importance of the nominated genes in PD, we examined whether they are differentially expressed in PD patients compared to controls, using expression data from single nuclear RNAseq (scRNA) from Kamath *et al*.^12^ and single nuclear and bulk RNAseq datasets from FOUNDIN-PD.^15^ Of the top nominated genes, *INPP5F* [average log fold-change (FC) = −7.22, *P* = 2.90 × 10^−31^] and *MLX* (average log FC = −1.80, *P* = 2.23 × 10^−4^) were associated with PD in the data published by Kamath *et al*.^12^ (Supplementary Table 6). In FOUNDIN-PD,^15^ after excluding prodromal cases, we found differential expression of many genes including *INPP5F* (average log FC = 0.070, *P* = 1.89 × 10^−19^) and *IP6K2* (average log FC = −0.076, *P* = 1.35 × 10^−35^) in scRNA data (*n* = 80) from dopaminergic neurons by comparing PD and controls (Supplementary Table 7). Results from the bulk RNAseq analysis of FOUNDIN-PD (*n* = 92) can be found in Supplementary Table 8.

### Structural analysis of *SPNS1* and *MLX*

Since non-synonymous variants in *SPNS1* and *MLX* were identified as major contributors to their selection as the nominated genes in their loci, we aimed to examine the potential consequences of these variants by performing *in silico* structural analyses of the protein encoded by these genes. *SPNS1* encodes a transporter for phospholipids at the lysosome membrane.^28^ It mediates the efflux of lysophosphatidylcholine and lysophosphatidylethanolamine out of the lysosome. The SNP rs7140 is located in the 3′-untranslated region (UTR) of the canonical splice variant 1 transcript, which produces the 528 amino acid (aa) isoform that has been investigated functionally^28^ (UniProt #Q9H2V7). This canonical isoform has also been observed in numerous proteomics datasets in gpmDB (https://gpmdb.thegpm.org/index.html). However, six other potential isoforms generated by alternative splicing have been predicted, including a 538 aa fragment with an alternative C-terminus, whereas the rs7140 SNP is located within the coding region (UniProt #H3BR82). The rs7140 variant results in the p.L512M mutation in this isoform. To investigate the impact of this mutation on the function of this *SNPS1* isoform, we inspected the 3D structure model generated by AlphaFold.^29^ Leu512 is located in the unstructured C-terminus of this membrane-bound protein, on the lumenal side of the lysosomal membrane (Supplementary Fig. 1A). The role of the C-terminus in this isoform of *SPNS1* remains unclear, and thus the impact of the p.L512M mutation is unknown.

The Max-like protein (MLX) is at the heart of a transcriptional network pathway involved in energy metabolism and cell signalling.^30,31^ It interacts with at least six other related proteins including the MAD family of transcriptional repressors and the Mondo family of transcriptional activators. These proteins contain basic/helix-loop-helix/leucine zipper (bHLHZ) domains that form heterodimers and interact with DNA carrying the CACGTG E-box motif. To understand the impact of the p.Q223R *MLX* mutation (rs665268) on its activity, we modelled the structure of MLX heterodimers with both the MAD and Mondo families using AlphaFold. MLX dimerizes with MAD1,^31^ and thus we superposed its bHLHZ domain on the MAD1-MAX-DNA complex crystal structure^32^ to generate the ternary complex model. The model shows that Gln223 in MLX is at the end of the dimerization ‘zipper’ helix (Supplementary Fig. 1B). The mutation p.Q223R induces the formation of a salt bridge with Glu139 in MAD1, which could strengthen the interaction between MAD1 and MAX. This could then downregulate the interaction of MAD1 with MAX through competition, and thus affect the extent of the transcriptional repression. Glu139 is not conserved in other MAD-related proteins such as MXI1 and MAD3/4. Furthermore, the model of MLX interacting with MLXIP, a protein of the Mondo family also known as MondoA,^33^ shows that the mutation may negatively affect the formation of this heterodimer by introducing a charge next to a hydrophobic sidechain (Supplementary Fig. 1C). The nuclear localization of Mondo proteins is dependent on their interaction with MLX,^30^ and thus the mutation may down regulate activation by the Mondo family while strengthening repression via MAD1.

### Gene enrichment analysis shows the inositol phosphate pathway as a novel pathway involved in PD

**Figure 6:**
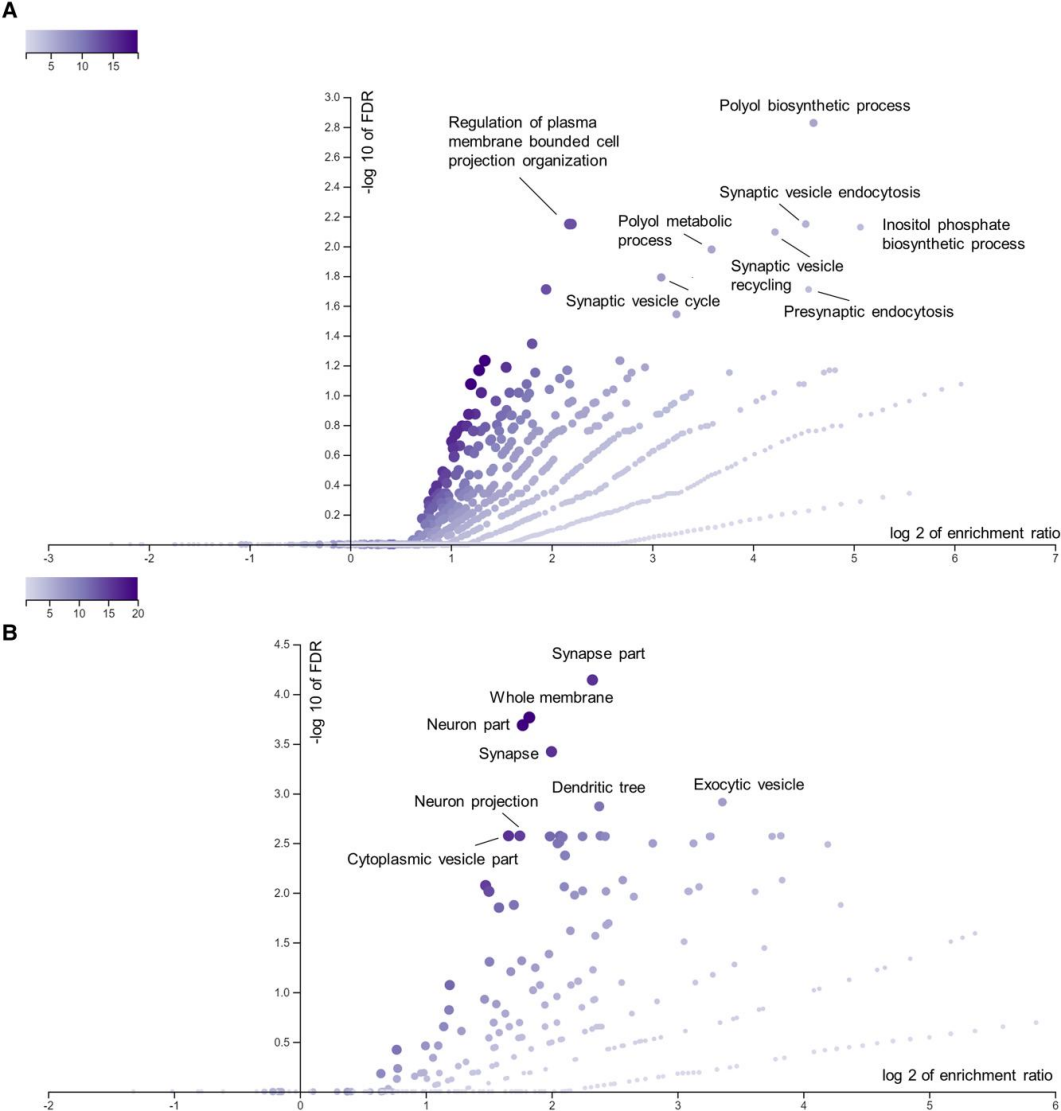
**Volcano plots of gene ontology biological processes and cellular components.** Volcano plots of gene-set enrichment analysis using WebGestalt showing the log of the false discovery rate (FDR) versus the enrichment ratio for biological processes (**A**) and cellular components (**B**). *P*-value are calculated using a hypergeometric test. All pathways that are significant after FDR correction were named.

We further examined whether the nominated genes highlighted specific pathways and mechanisms associated with PD. We performed a pathway enrichment analysis by examining over-representation of the top nominated genes in biological processes and cellular components using the top genes in each locus. Among the biological processes passing the FDR correction, the inositol phosphate biosynthetic process (GO:0032958) and polyol biosynthetic process (GO:0046173) were strongly enriched (Fig. 6A). Inositol was associated with four candidate genes, namely *ITPKB*, *IP6K2*, *PPIP5K2* and *INPP5F*. The features most important to the nomination of these genes as PD-associated by our ML model are shown in Fig. 5. Cellular components were also identified in the gene enrichment analysis (Fig. 6B).

### Pathway-specific polygenic risk score of the inositol phosphate pathway is associated with PD

To further study the association between the putative novel PD pathways and PD status, pathway-specific PRSs were calculated for the above-mentioned gene sets. The association between these PRSs and PD was examined in six PD cohorts, followed by a meta-analysis as detailed in the ‘Materials and methods’ section. One outlier cohort was excluded due to heterogeneity. The pathway-specific PRSs were first calculated using all genes in that pathway. Then, to further validate that the specific pathway was indeed important in PD, we excluded the genes nominated by our machine learning pathway and recalculated the PRS. By removing these genes with GWAS significant signals, we could examine the residual effect of the remaining pathway. The inositol phosphate biosynthetic pathway was associated with PD even after excluding the genes nominated in our analysis [odds ratio (OR) 1.06, 95% confidence interval (CI) 1.03–1.09, *P* = 7.01 × 10^−5^], as well as other related pathways (Table 1). Forest plots of the all the pathway PRSs are shown in Supplementary Fig. 2.

**Table 1:** Meta-analyses of pathway-specific polygenic risk scores

Pathway-specific polygenic risk score	OR	95% CI	*P*-value	Het *P*-value
POLYOL_BIOSYNTHETIC_PROCESS	1.20	1.17–1.24	2.07 × 10^−42^	1.91 × 10^−5^
INOSITOL_PHOSPHATE_BIOSYNTHETIC_PROCESS	1.15	1.12–1.18	2.36 × 10^−25^	1.97 × 10^−2^
POLYOL_BIOSYNTHETIC_PROCESS_filtered	1.09	1.06–1.12	1.04 × 10^−9^	1.12 × 10^−2^
INOSITOL_PHOSPHATE_BIOSYNTHETIC_PROCESS_filtered	1.06	1.03–1.09	1.31 × 10^−5^	1.45 × 10^−1^

CI = confidence interval; Filtered = excluded Parkinson’s disease genome-wide association study top gene; GOBP_INOSITOL_PHOSPHATE_BIOSYNTHETIC_PROCESS = Gene Ontology inositol phosphate biosynthetic process (GO:0032958); GOBP_POLYOL_BIOSYNTHETIC_PROCESS = Gene Ontology polyol biosynthetic process (GO:0046173); Het = heterogeneity; OR = odds ratio.

### Rare *KCNIP3* and *LSM7* variants and in the polyol/inositol biosynthetic pathway are involved in PD

To further establish the potential role of the nominated genes in PD, we performed rare variant burden tests in all the genes nominated by our model. As expected, genes that are known to harbour rare PD coding mutations including *GBA1*, *LRRK2* and *GCH1* were associated with PD (Table 2 and Supplementary Table 9). Three additional genes, including two genes that have not previously been implicated in PD (*KCNIP3* and *LSM7*) showed a burden of rare variants after FDR correction for multiple comparisons. We then examined the genes from the pathway enrichment analysis and found that rare variants in the polyol/inositol biosynthetic pathway were also associated with PD (SKAT-O, *P* = 1.58 × 10^−4^), further supporting its role in PD.

**Table 2:** Meta-analysis of rare variant analysis of putative causal genes

Set	*P*-value	FDR *P*-value
*GBA1* Rarefunctional	2.04 × 10^−12^	6.22 × 10^−10^
*GBA1* Rarenonsyn	3.38 × 10^−11^	5.15 × 10^−9^
*GBA1* RareLOF	1.22 × 10^−6^	1.24 × 10^−4^
*GBA1* RareCADD	2.32 × 10^−6^	1.77 × 10^−4^
*LSM7* RareLOF	3.69 × 10^−6^	2.25 × 10^−4^
*KCNIP3* RareLOF	1.12 × 10^−5^	5.69 × 10^−4^
*GCH1* RareLOF	2.02 × 10^−5^	8.80 × 10^−4^
*LRRK2* RareCADD	6.07 × 10^−5^	2.31 × 10^−3^
Polyol Rarefunctional	1.59 × 10^−4^	5.38 × 10^−3^
Polyol Rarenonsyn	2.86 × 10^−4^	8.74 × 10^−3^
*NUCKS1* RareCADD	4.13 × 10^−4^	1.14 × 10^−2^
Polyol RareLOF	1.54 × 10^−3^	3.91 × 10^−2^
*SYT17* Rarenonsyn	4.61 × 10^−3^	9.37 × 10^−2^
*P2RY12* RareLOF	4.38 × 10^−3^	9.37 × 10^−2^
*CYLD* RareLOF	4.48 × 10^−3^	9.37 × 10^−2^
*SYT17* Rarefunctional	7.39 × 10^−3^	1.38 × 10^−1^
*LCORL* RareLOF	7.66 × 10^−3^	1.38 × 10^−1^
*CAMK2D* RareLOF	8.62 × 10^−3^	1.46 × 10^−1^
*FBRSL1* RareLOF	1.12 × 10^−2^	1.80 × 10^−1^
*CTSB* RareLOF	1.20 × 10^−2^	1.82 × 10^−1^
*KPNA1* RareCADD	1.35 × 10^−2^	1.96 × 10^−1^
*ASXL3* RareLOF	1.52 × 10^−2^	2.10 × 10^−1^
*KPNA1* RareLOF	1.76 × 10^−2^	2.33 × 10^−1^
*LRRK2* Rarefunctional	2.57 × 10^−2^	3.14 × 10^−1^
*MICU3* RareLOF	2.56 × 10^−2^	3.14 × 10^−1^
*VAMP4* Rarenonsyn	2.93 × 10^−2^	3.43 × 10^−1^
*MBNL2* RareCADD	3.04 × 10^−2^	3.43 × 10^−1^
*LRRK2* Rarenonsyn	3.28 × 10^−2^	3.57 × 10^−1^
*KPNA1* Rarefunctional	3.46 × 10^−2^	3.64 × 10^−1^
*LSM7* Rarefunctional	3.58 × 10^−2^	3.64 × 10^−1^
*HIP1R* Rarenonsyn	3.93 × 10^−2^	3.87 × 10^−1^
*KPNA1* Rarenonsyn	4.23 × 10^−2^	3.91 × 10^−1^
*HIP1R* Rarefunctional	4.22 × 10^−2^	3.91 × 10^−1^

FDR = false discovery rate; Rarefunctional = rare functional variants; Rarenonsyn = rare non-synonymous variants; RareLOF = rare loss-of-function variants; RareCADD = rare variants with CADD score > 20; Set = variant set across genes/pathway.

## Discussion

Using multi-omic data and machine learning, we nominated genes that potentially drive the associations with PD for each of the 78 PD GWAS loci. Our nominated genes included many not previously studied in the context of PD. Additionally, we identified two novel genes with rare variants (*KCNIP3* and *LSM7*) as well as genes with GWAS significant coding variants such as *SPNS1* and *MLX* that could be further studied. Furthermore, our gene enrichment, pathway-specific PRS and rare variant analyses suggested involvement of the inositol phosphate biosynthetic pathway in PD.

Four genes nominated by our machine learning model were associated with the inositol phosphate biosynthetic pathway, *ITPKB*, *IP6K2, PPIP5K2* and *SNCA*,^34^ which showed strong enrichment of this pathway. In addition, *INPP5F*, also nominated by our analysis, is involved in inositol processing through a parallel pathway.^35^ Our results demonstrate that the inositol pathway-PRS, even when excluding the previously mentioned genes, is still associated with PD. Taken together, our findings support the importance of the inositol phosphate pathway in PD.

Based on the evidence from the candidate inositol genes and previous inositol studies, inositol could potentially be a therapeutic target for PD. In 1999, a clinical trial on inositol was conducted on nine PD patients.^36^ Treatment with inositol compared with placebo did not improve clinical outcomes; however, we cannot rule out inositol and inositol phosphates as potential therapeutic targets, as only nine patients were recruited for the trial.

*ITPKB* encodes for a ubiquitous kinase that phosphorylates inositol 14,5-trisphosphate (IP3) to inositol 1,3,4,5 tetrakisphosphate (IP4) using a Ca^2+^/calmodulin-dependent mechanism. IP3 is a secondary messenger that stimulates calcium release from the endoplasmic reticulum (ER). In primary neurons, *ITPKB* knockdown/overexpression was shown to increase/reduce levels of α-synuclein aggregation.^37^ Additionally, *ITPKB* knockdown in neurons leads to the accumulation of calcium in mitochondria. This accumulation can impair the process of autophagy, which is crucial for maintaining mitochondrial health. In neuroblastoma cells, *ITPKB* mRNA levels were also shown to be correlated with *SNCA* expression in the cortex and *IPTKB* protein levels were increased in wild-type α-synuclein, A53T and A30P mutants.^38^ Meanwhile, *IP6K2* and *PPIP5K2* interact with the same substrates. *IP6K2* converts inositol hexakisphosphate (IP6) to 5-diphosphoinositol pentakisphosphate (5-IP7) or 1-diphosphoinositol pentakisphosphate (1-IP7) to bis-diphosphoinositol tetrakisphosphate (1,5-IP8), while *PPIP5K2* convert 5-IP7 to 1,5-IP8 and IP6 to 1-IP7.^39^ In mice, *IP6K2* has been implicated in cell death, apoptosis and neuroprotection.^40^ One study proposed that *IP6K2* regulates mitophagy via the parkin/PINK1 pathway, but further evidence would be required to confirm this hypothesis.^40^*PPIP5K2* has not previously been implicated in PD but is associated with hearing loss and colorectal carcinoma.^41,42^ Finally, *INPP5F* is involved with a different inositol pathway; it encodes SAC2, which converts phosphoinositides such as PI(4,5)P2 to phosphatidylinositol during endocytosis.^35^

Inositol phosphate has been suggested to be involved in obesity, insulin resistance and energy metabolism.^43^ In post-mortem brain tissues of PD patients, ^3^H-inositol 14,5-trisphosphate binding sites were found to be reduced in certain brain regions such as the caudate nucleus, putamen and pallidum.^44^ Additionally, IP6 was shown to be associated with PD. IP6 has a neuroprotective effect on dopaminergic cells by preventing 6-OHDA-induced apoptosis.^45^ IP6 inhibits the activity of β-secretase 1 (BACE1), an enzyme that cleaves amyloid-β precursor protein into toxic amyloid-β peptides.^46^ Paraquat-induced neurodegeneration in *Drosophila* was suggested to increase the levels of inositol phosphates metabolites.^47^ Previous studies have also suggested that different stereoisomers of inositol such as *scyllo*-inositol can inhibit the aggregation of α-synuclein^48^ or decrease the myoinositol concentration in patients with PD.^49,50^

Recent studies on inositol investigated the role of *SYNJ1*, an autosomal recessive form of early-onset parkinsonism.^51^ SYNJ1 is a lipid phosphatase of phosphatidylinositol-34,5-trisphosphate (PIP3).^52^ SYNJ1 knockout cell models were associated with an increase of α-synuclein and PIP3 levels. PIP3 dysregulation was suggested to promote α-synuclein aggregation, which increases the risk of PD. Together with our data, there is strong evidence for the involvement of the inositol phosphate biosynthetic pathway in PD, and this pathway should be further studied using both basic science and translational approaches.

Outside of the inositol pathway, *SPNS1* and *MLX* were found to be the top causal gene in their respective loci with putative causal missense SNPs: rs7140 and rs665268. Rs7140 corresponds to p.Leu563Val on the *SPNS1* transcript variant X1. We found that *SPNS1* expression is lower in the SOX6_ATGR1 dopaminergic neuron subpopulation in PD compared with controls. This subcluster was previously highlighted to be the most susceptible to neurodegeneration in PD.^12^*S*PNS1 encodes a sphingolipid transmembrane transporter in the lysosome. The autophagy-lysosomal pathway has been well-established to be crucial in PD pathogenesis, especially the lysosomal sphingolipid metabolism pathway, which includes well established PD-associated genes including GBA1, GALC, SMPD1 and others.^53,54^ SPNS1 deficiency results in lipid accumulation in the lysosome and impaired lysosomal function.^28^

The second nominated gene in which we identified rare variants, *MLX*, encodes a Max-like protein X which belongs to a family of transcription factors regulating glucose metabolism. Rs665268 is a missense variant (p.Gln139Arg) that was found to be associated with Takayasu’s arteritis, an autoimmune systemic vasculitis.^55^*MLX* was also reported to be associated with age at onset of Alzheimer’s disease in females.^56^ This variant was suggested to affect two important PD pathways by increasing oxidative stress and suppressing autophagy in immune cells.^55,56^*SPNS1* and *MLX* have not previously been implicated in PD. Both variants, rs7140 and rs665268, were found in high LD with the top candidate GWAS SNP. When examining missense SNPs in LD with the top GWAS SNPs, *SPNS1*, *MLX* and *CD19* were the only genes with such features. *CD19* was not nominated in our study, as it is located in the same locus as *SPNS1*, and it ranked lower than *SPNS1*. These findings indicate that these genes could play a role in PD and should be further studied.

Other studies have attempted to use machine learning to characterize genes involved in PD. Using machine learning, Ho *et al*.^57^ integrated tissue-specific eQTLs and the genotypes of PD patients and controls to identify PD-specific genes. They nominated the roles of two key variants in PD (rs7617877, rs6808178) and the potential role of heart atrial appendage tissue. Interestingly, *AGTR1*, a gene associated with many PD single-nuclei subpopulations included in our model, encodes for angiotensin II receptor type 1 protein.^58^ This protein is part of the renin-angiotensin system, which regulates blood pressure and the balance of fluids and salts in the body.^58^ Ho *et al*.^57^ also validated some of the top genes from our model such as *INPP5F*, *P2RY12*, *HIP1R*, *STK39* and *CTSB*. Transcriptional changes to these genes could contribute to PD.

Interestingly, in certain regions of the genome, such as *VPS13C*, the top genes showed lower probability scores (Fig. 2). This could be due to complex LD structure, which weakens the effect size of eQTLS, as the variants in LD are associated with multiple genes. In such scenarios, the model might encounter challenges in precisely predicting the responsible gene. Additionally, the number of samples employed in statistically assessing attributes like eQTLs and enhancer-promoter interactions significantly impacts the model’s training. Features derived from studies with limited sample sizes may be less powered to detect eQTLs and more likely to be excluded from the model. For example, while data regarding enhancer-promoter interactions were incorporated into the training attributes, it might not have been important for the majority of variant-gene pairs. Overall, while *VPS13C* had a low probability score for a gene in the training set, it was still the top gene in its respective locus.

Although we identified candidate genes and new rare mutations, there were several limitations to this study. This study was based on a GWAS of European populations only. Therefore, our results are potentially restricted to Europeans. While there are some studies on the association of chromosome X in PD, the statistical power was limited compared with a PD GWAS of autosomes. As a result, no analysis was performed on chromosome X. In addition, the training set for the machine learning model was limited to a small set of known or highly likely PD genes with the assumption of one causal gene per locus. The study also lacked samples for a testing set due to the small number of well-established PD genes. Since these limitations may have introduced some bias, we used different strategies such as controlling for an imbalanced dataset and choosing balanced accuracy as an evaluation function to maximize the performance of the model. Although the distance between variants and genes holds significant predictive power in the model, it is crucial to acknowledge that not all top genes can accurately be predicted solely based on distance. Of the 78 genes analysed, 13 were not the closest genes in terms of distance from the gene to the top GWAS SNPs, and 25 were not the closest genes based on distance to the transcription start site. Additionally, when comparing the scRNA and bulk RNAseq results, most of the differentially expressed genes did not overlap across our datasets. For example, while *INPP5F* was nominated in scRNA of both datasets, it was not significant in the bulk RNAseq analysis. Lastly, the meta-analysis of rare variants can also be somewhat biased due to case/control imbalance. Larger GWAS and functional studies will be required to validate our findings.

Our results nominated multiple genes that have not been thoroughly studied in PD and provide a foundation for future functional studies of these genes. As larger PD GWASs will nominate more SNPs and loci, prioritizing causal genes will be crucial to understanding the underlying biological mechanisms and disease pathophysiology through additional studies. Future gene prioritization studies will be able to leverage larger datasets with more positive labels as new PD genes are discovered, increasing the accuracy of predictions.

## Supplementary Material

awad345_Supplementary_Data

## Data Availability

The data used for this study can be accessed on: AMP-PD (https://amp-pd.org/); Bryois *et al*.^59^; Cuomo *et al*.^60^; FUMA https://fuma.ctglab.nl/; Kamath *et al.*^12^; PPMI ppmi-info.org; SMR https://yanglab.westlake.edu.cn/software/smr/and UK Biobank https://www.ukbiobank.ac.uk/. The scripts used for this study can be found on GitHub: github.com/gan-orlab/gene_prio.
