# Supplementary material for: Machine learning nominates the inositol pathway and novel genes in Parkinson’s disease
Source: Brain. 2023 Oct 6;147(3):887–99. doi: 10.1093/brain/awad345 (PMC10907089; doi:10.1093/brain/awad345)
Supplement: awad345_Supplementary_Data [file awad345_supplementary_data.zip › brain-2023-01287-File011.pdf]

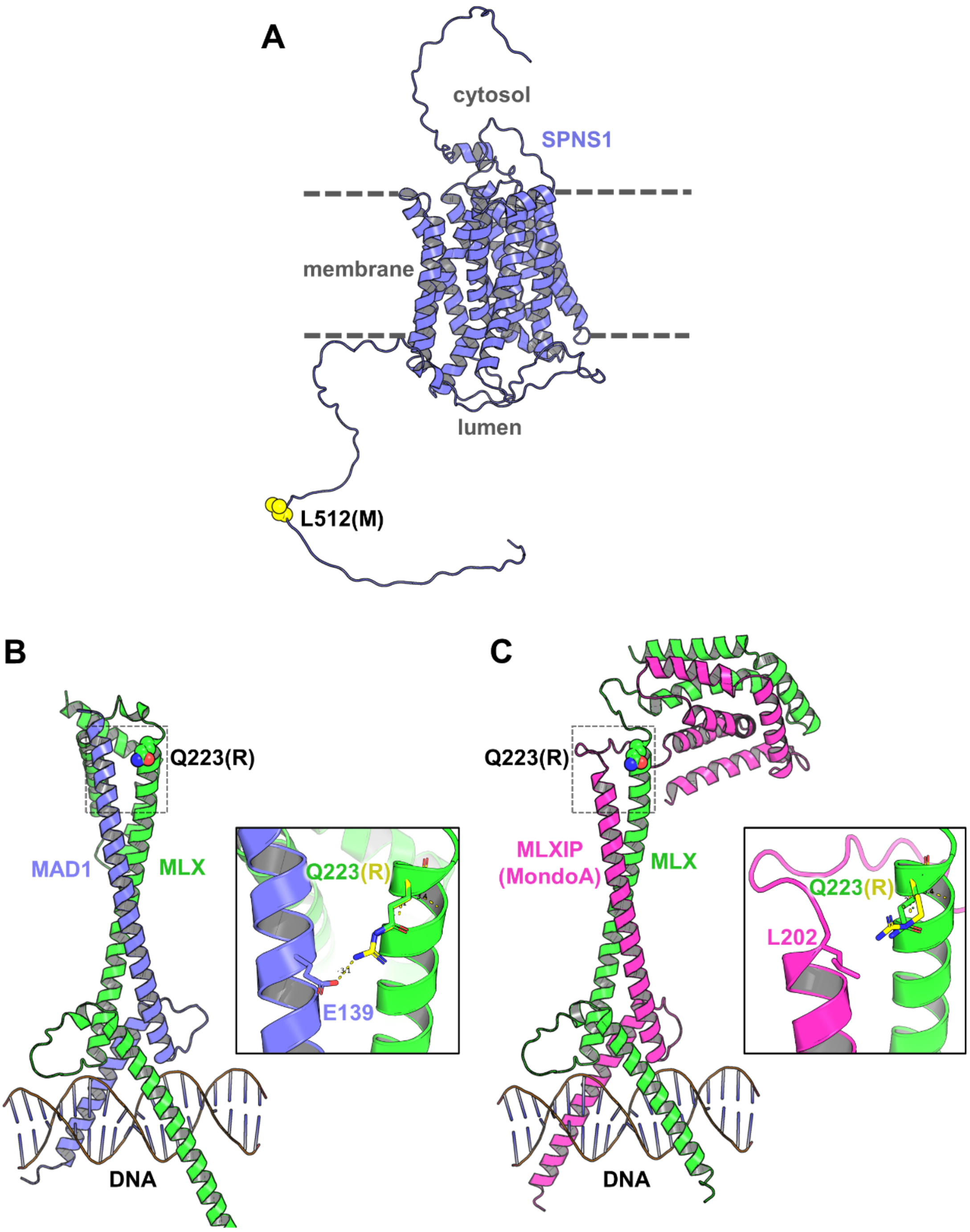

Supplementary Figure 1 Structural analysis of SPNS1 p.L512M and MLX p.Q223R. (A) Alphafold prediction of the structure of the lysophospholipid transporter SPNS1 (alternative isoform, Uniprot #H3BR82). The mutation p.L512M would take place in the lumen of the lysosome. (B) AlphaFold model of the MAD1-MLX heterodimer superposed on the structure of the MAD1-MAX-DNA complex (PDB 1NLW). The inset is a zoom on the MLX p.Q223R mutation, displaying the effect that the mutation may have on the interaction with the MAD1 protein. C) AlphaFold model of the MLXIP-MLX heterodimer superposed on the structure of the MAD1-MAX-DNA

complex, as described above. Note that AlphaFold also predicts an interaction between the C-termini of MLXIP and MLX (but not MAD1 and MLX).

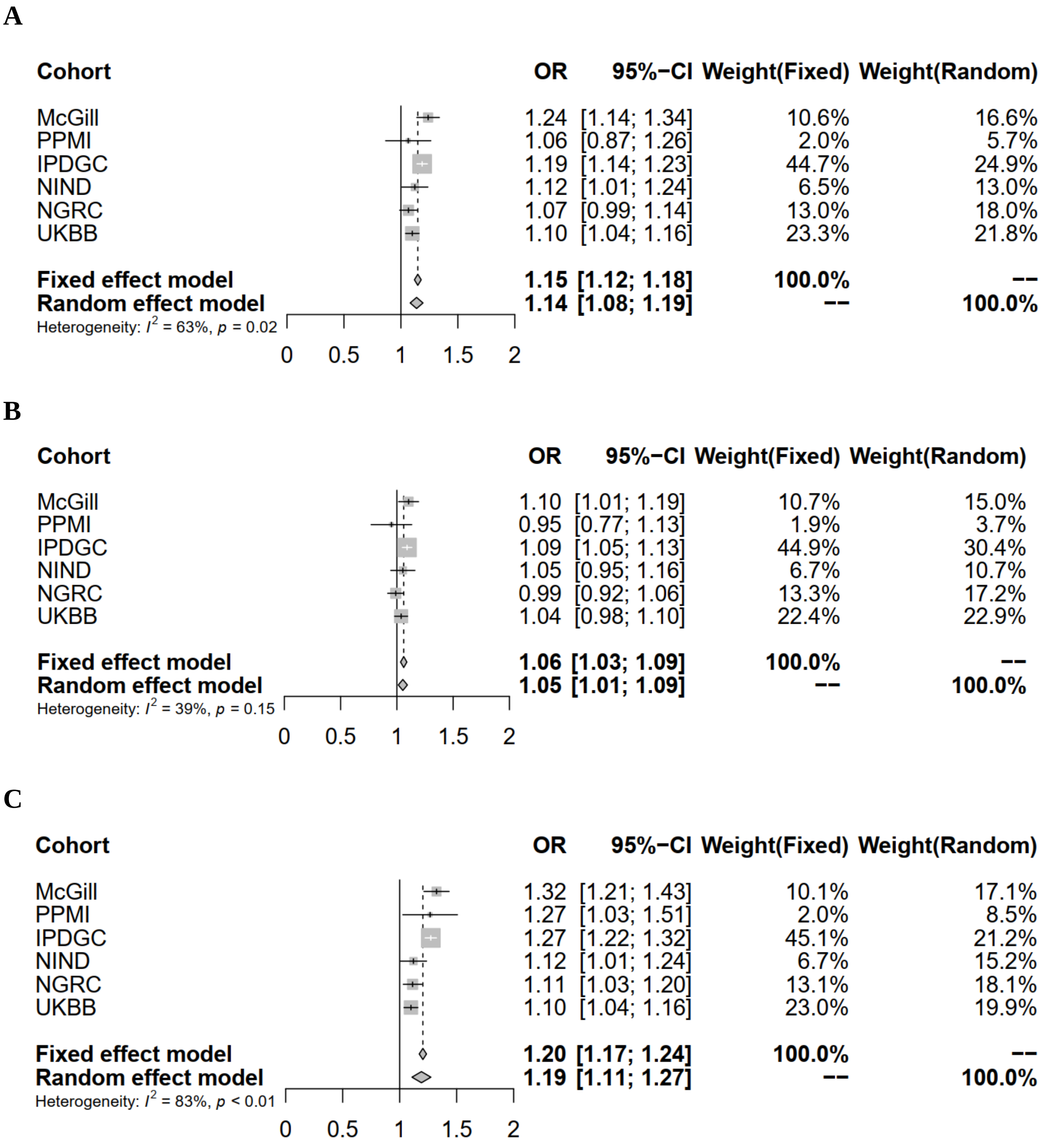
A
Cohort OR 95%−CI Weight(Fixed) Weight(Random)
McGill 1.24 [1.14; 1.34] 10.6% 16.6%
PPMI 1.06 [0.87; 1.26] 2.0% 5.7%
IPDGC 1.19 [1.14; 1.23] 44.7% 24.9%
NIND 1.12 [1.01; 1.24] 6.5% 13.0%
NGRC 1.07 [0.99; 1.14] 13.0% 18.0%
UKBB 1.10 [1.04; 1.16] 23.3% 21.8%
Fixed effect model 1.15 [1.12; 1.18] 100.0% --
Random effect model 1.14 [1.08; 1.19] -- 100.0%
Heterogeneity: I^2 = 63%, p = 0.02
0 0.5 1 1.5 2
B
Cohort OR 95%−CI Weight(Fixed) Weight(Random)
McGill 1.10 [1.01; 1.19] 10.7% 15.0%
PPMI 0.95 [0.77; 1.13] 1.9% 3.7%
IPDGC 1.09 [1.05; 1.13] 44.9% 30.4%
NIND 1.05 [0.95; 1.16] 6.7% 10.7%
NGRC 0.99 [0.92; 1.06] 13.3% 17.2%
UKBB 1.04 [0.98; 1.10] 22.4% 22.9%
Fixed effect model 1.06 [1.03; 1.09] 100.0% --
Random effect model 1.05 [1.01; 1.09] -- 100.0%
Heterogeneity: I^2 = 39%, p = 0.15
0 0.5 1 1.5 2
C
Cohort OR 95%−CI Weight(Fixed) Weight(Random)
McGill 1.32 [1.21; 1.43] 10.1% 17.1%
PPMI 1.27 [1.03; 1.51] 2.0% 8.5%
IPDGC 1.27 [1.22; 1.32] 45.1% 21.2%
NIND 1.12 [1.01; 1.24] 6.7% 15.2%
NGRC 1.11 [1.03; 1.20] 13.1% 18.1%
UKBB 1.10 [1.04; 1.16] 23.0% 19.9%
Fixed effect model 1.20 [1.17; 1.24] 100.0% --
Random effect model 1.19 [1.11; 1.27] -- 100.0%
Heterogeneity: I^2 = 83%, p < 0.01
0 0.5 1 1.5 2

D

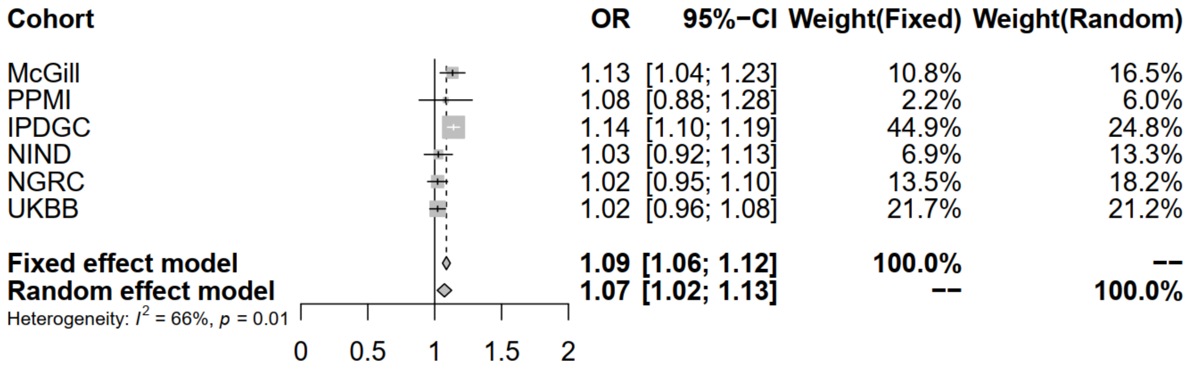

Supplementary Figure 2: Forest plot of meta-analysis of pathway-specific polygenic risk score. (A) Inositol phosphate biosynthetic process (GO:0032958) (B) Inositol phosphate biosynthetic process (GO:0032958) excluding Parkinson’s disease GWAS loci candidate gene (C) Polyol biosynthetic process (GO:0046173) (D) Polyol biosynthetic process (GO:0046173) excluding Parkinson’s disease GWAS loci candidate gene
